# The Asia 2 specific signal peptide region and other domains in fusion protein genes characterized Asia 1 and Asia 2 canine distemper viruses

**DOI:** 10.1186/1743-422X-6-157

**Published:** 2009-10-06

**Authors:** Serageldeen Sultan, Nataya Charoenvisal, Nguyen Thi Lan, Ryoji Yamaguchi, Ken Maeda, Kazushige Kai

**Affiliations:** 1 Department of Veterinary Microbiology, Faculty of Agriculture, Yamaguchi University, Yamaguchi 753-8515, Japan; 2 Department of Veterinary Pathology, Faculty of Agriculture, University of Miyazaki, Miyazaki 889-2192, Japan; 3 Department of Veterinary Pathology, Faculty of Veterinary Medicine, Hanoi University of Agriculture, Trau Quy-Gia Lam-Ha Noi, Vietnam

## Abstract

**Background:**

Although the presence of Asia 2 group of canine distemper virus (CDV) was known by the sequencing and phylogenetic analysis of hemagglutinin (H) gene, the fusion (F) protein gene sequence of Asia 2 group had not been identified. So, the sequence analysis of F gene was carried out to elucidate the genotypic varaitons among Asian isolates.

**Results:**

The phylogenetic analysis of F and H gene sequences from fourteen CDV isolates obtained from diseased dogs in Japan and Thailand indicated that the F genes had a new initiation codon and extra 27 nucleotides upstream of the usual open reading frame (ORF) and the F proteins had extra 9 amino acids at the N-terminal position only in Asia 2 isolates. On the contrary, the Asia 1 isolates had three extra putative N-glycosylation sites (two sites in the signal peptide region and one site in the F1 region) except for two strains of Th12 and Ac96I (two sites in signal peptide region) adding to four putative N-glycosylation sites that were conserved among all Asian isolates and Onderstepoort strain. In addition to this difference in N-glycosylation sites, the signal peptide region had a great diversity between Asia 1 and Asia 2 isolates. Also, characteristic amino acids were detected for some strains.

**Conclusion:**

Asia 2 isolates were distinguished from other CDV lineages by the extra 27 nucleotide sequence. The signal peptide region of F gene gives a remarkable differentiation between Asia 1 and Asia 2 isolates. Strains Th12 and Ac96I were differentiated from other Asia 1 strains by the F protein glycosylation sites.

## Background

Canine distemper virus (CDV) is a single strand RNA virus belonging to genus *Morbillivirus *within the family *Paramyxoviridae*. The CDV genome encodes the following virion proteins: nucleocapsid (N), phosphoprotein (P), matrix (M), fusion (F), hemagglutinin (H) and polymerase (L). The F protein mediates pH-independent fusion of the viral envelope with the plasma membrane of the host cell [1].

Paramyxovirus fusion proteins are synthesized as an inactive precursor F0 that is cleaved by a host-cell protease to release the new N-terminus of the F1 [2]. Thus, forming the biologically active protein consists of the disulfide linked chain F1 and F2 [3]. The membrane anchored F1 subunit contains several regions important for promotion of membrane fusion. At its C-terminus, a hydrophobic trans-membrane domain (TM) anchors the protein in the membrane leaving a short cytoplasmic tail (20-40 residues). The fusion peptide, locates at the F1 subunit N-terminus, has been demonstrated to insert into the target membrane upon initiation of membrane fusion [4]. Also, F1 contains two heptad repeat regions, one close to C-terminal of the fusion peptide (HRA) and the other adjacent to the trans-membrane domain (HRB) [2,5,6]. To date, intensive studies were carried out on the H gene sequencing and phylogenetic relationship analysis [7-14] but a little is known about the F gene variations. Two genotypes of H gene, Asia 1 [9,15] and Asia 2 [12], have been recognized among Asian isolates of CDV and they were found to differ from those of the European and American CDV genomes. In this study the phylogenetic characterization of F as well as H protein genes among Asian isolates of CDV was carried out to know the genetic variations of F genes.

## Results

### Phylogenetic analysis of deduced amino acids of H genes

**Figure 1:**
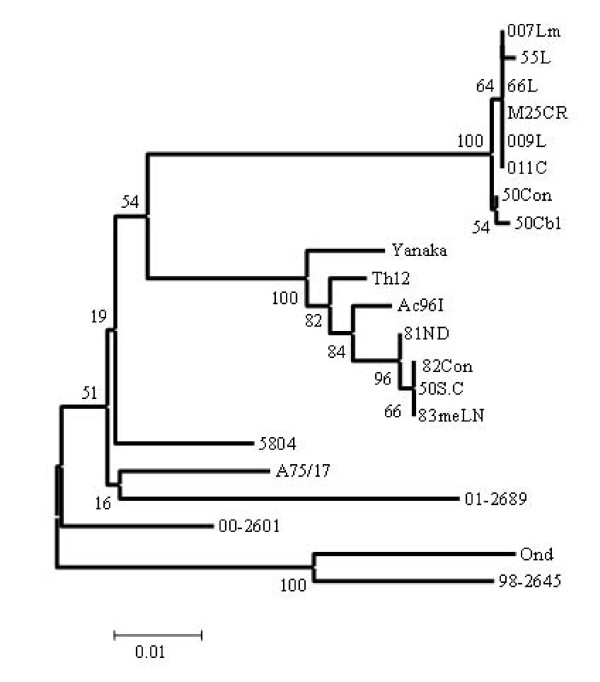
**Phylogenetic analysis of deduced amino acid sequences of H gene of Asian isolates of canine distemper virus using the neighbor-joining method in Mega 3.1 program**. Accession numbers of CDV used for comparison are shown in parentheses as follows: A75/17 (AF164967), 5804 (AY386315), 00-2601 (AY443350), 01-2689 (AY649446), 98-2645 (AY445077), Yanaka (D87949) and Onderstepoort (AF378705).

The phylogenetic relationship based on the deduced amino acid sequences of the H protein of fourteen CDV strains were analyzed as shown in Fig. 1. As a result, strains 007Lm, 55L, 66L, 009L, M25CR, 011C, 50Con and 50Cbl were classified into Asia 2 group and strains Ac96I, Th12, 50Sc, 81ND, 82Con and 83mLN were classified into Asia 1 group. Among the Asia 2 strains, 007Lm, 66L, 009L, M25CR, and 011C had identical amino acid sequences of the H gene, although strain 009L differed from strains 007Lm, 66L, M25CR, and 011C in its nucleotide sequence of the H gene (99% identity). In addition, these four strains differed from strains 55L, 50Con, and 50 Cb1 in both amino acid and nucleotide sequences (99% identity of both amino acid and nucleotide sequences). On the other hand, among the Asia 1 strains, 50Sc, 82Con, and 83mLN had identical amino acid sequences of the H gene although strain 82Con differed from strains 50Sc and 83mLN in the nucleotide sequences of the H gene (99% identity). However, these three strains differed from strains Ac96I, Th12, and 81ND in both amino acid (identity 99% with Ac96I and 81ND, and 98% with Th12) and nucleotide sequence (identity 99% with Ac96I and 81ND, and 98% with Th12).

### Extra 27 nucleotides upstream of the usual F gene initiation codon characterized Asia 2 strains

**Figure 2:**
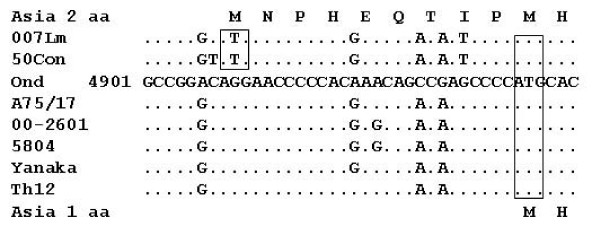
**Alignment of the nucleotide sequences of different CDV field isolates and Onderstepoort strain from nt 4901 to 4940 upstream of the usual initiation codon of F gene ORF (start from nt 4935) showing the extra 27 nucleotides and their deduced 9 amino acids in Asia 2 isolates**. The following strains had identical sequences; Asia 2 strains (007Lm, 55L, 66L, 009L, M25CR and 011 C), (50Con and 50Cbl), Asia 1 strains (Th12, Ac96I, 50Sc, 81ND, 82Con and 83mLN) and American strains (98-2645, 00-2601 and 01-2689). Accession numbers as in Fig. 1.

Sequence analyses of the F gene revealed a new initiation codon and extra 27 nucleotides upstream of the usual F gene open reading frame (ORF) in all Asia 2 isolates. To characterize this nucleotide sequence, which extended from 4908 to 4934, various CDV strains as well as the present fourteen strains were compared about the nucleotide sequences from 4901 to 4940 as shown in Fig. 2. Interestingly, only Asia 2 isolates have a nucleotide change from ^4909^G to ^4909^T which led to the expansion of the F ORF. All Asia 2 isolates (007Lm, 55L, 66L, 009L, M25CR, 011C, 50Con and 50Cbl) had an identical 27 nucleotide sequence. In addition, other nucleotide differences were found among Asia 2 isolates such as ^4907^T/^4907^C that characterized 50Con and 50Cbl from other strains, also ^4920^G/^4920^A and ^4930^T/^4930^G characterized all Asia 2 strains although American and European as well as Yanaka (Asia 1) strains have the same nucleotide at position 4920 as Asia 2 isolates. The ^4926^A and ^4928^A were shared by all strains in compared to Onderstepoort strain (Fig. 2).

### Structure of the F gene product and cleavage sites stability

**Figure 3:**
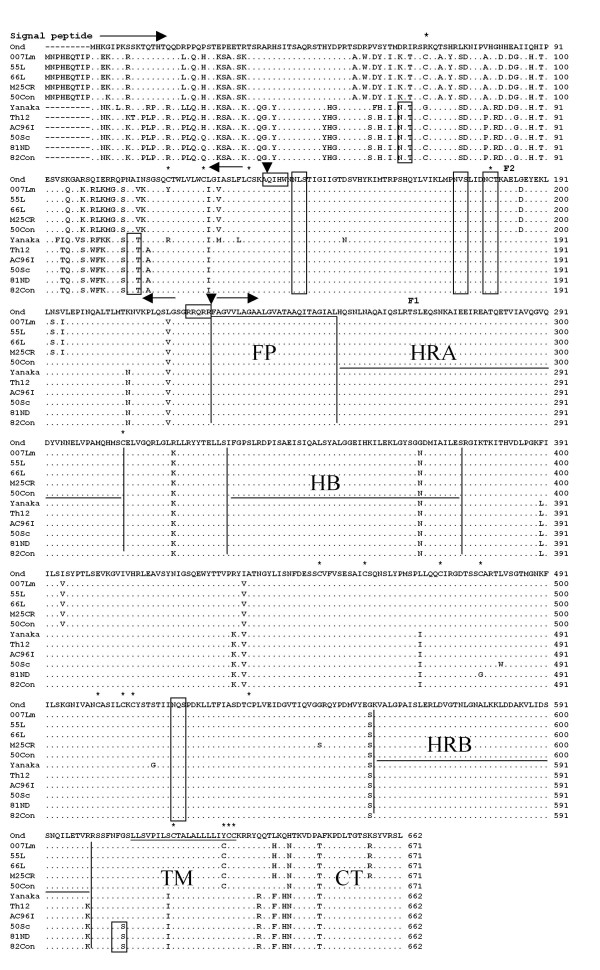
**Alignment of deduced amino acid sequences of F genes of CDV strains**. Only amino acids differ from the Onderstepoort sequence are shown. Potential N-linked glycosylation sites (N-X-S/T) are boxed. Cysteine residues (*), cleave sites (▼), Hydrophobic regions are underlined. Domains in the F gene are Fusion peptide (FP), heptad repeats (HRA and HRB), helical bundles (HB), trans-membrane (TM), cytoplasmic tail (CT). Numbering starts at the first methionine residue of the Onderstepoort strain. The predicted amino acid sequences of the following pairs were identical; 66L/009L, M25CR/011C, 50Con/50Cbl and 82Con/83mLN.

The F genes of fourteen Asian CDV isolates were sequenced and the deduced amino acids were aligned to detect the genetic variations among Asian isolates as shown in Fig. 3. The F gene product is cleaved by cellular proteases of signal peptidase and furin into three regions; signal peptide, F2 and F1 [2]. The cleavage sites, AQIHW in the C-terminus of signal peptide region and RRQRR in the N-terminus of F1 region [16-18], were highly conserved in all Asian isolates as shown in Fig. 3.

### Signal peptide region

The signal peptide region is an important region for location of the precursor F0 into golgi network to cleave into F1 and F2 for fusion activity [19] and is a highly divergent region [2]. As shown in Fig. 3 all Asia 2 isolates have extra 9 amino acids upstream of the N-terminus of this region which is a characteristic for Asia 2 isolates. The amino acid variations were 30 - 32 % and 34 - 35 % while the nucleotide differences were 15 - 16 % and 18 - 19 % for Asia 2 and Asia 1, respectively in comparison with the Onderstepoort strain in the signal peptide region. Both Asia 1 and Asia 2 isolates have common amino acids which differ from Ondestepoort. Moreover, each group has its specific amino acids. However, within the same group there is amino acid(s) characteristic to individual strain as in 007Lm ^116^Y/^116^C and inTh12 ^9^T/^9^S and ^26^R/^26^K (Fig. 3).

### F2 and F1 regions

In the F2 region (aa136-224), amino acid differences were found as ^208^N/^208^K and ^216^V/^216^L in Asia 1 isolates, whereas ^186^D/^186^G, ^193^S/^193^N, ^195^V/^195^I and ^216^V/^216^L in Asia 2 isolates but strains 50Con and 50Cbl have the same amino acid at positions 193 and 195 as Onderstepoort strain (Fig. 3).

The membrane anchored F1 subunit contains the fusion peptide (FP) domain (hydrophobic) at the N-terminus, trans-membrane (TM) domain (hydrophobic) and the cytoplasmic tail (CT) domain at the C-terminus. The fusion peptide domain was highly conserved among all CDV strains. On the other hand, amino acid changes were found in the TM domain as ^616^I/^616^S in all Asia 1 isolates and ^627^C/^627^Y in all Asia 2 isolates. In the CT domain, six amino acid changes were observed within a span of 33 amino acid sequence. Common amino acid changes in all Asian isolates were found as ^640^N/^640^H and ^646^T/^646^A. Specific amino acid changes to all Asia 1 isolates were found as ^634^R/^634^Q, ^637^F/^637^L and ^639^H/^639^Q while those specific to Asia 2 isolates were found as ^637^H/^637^L and ^656^R/^656^K. Strains 50Con and 50Cbl had the same amino acids at positions 637 and 656 as Onderstepoort strain.

Adjacent to these domains, heptad repeats were designated as HRA (aa 250-307), HB (aa 328-374) and HRB (aa 557- 601), respectively [6,20,21] as shown in Fig. 3. Amino acid changes were found in HB domain as ^366^N/^366^G in all Asian isolates and as ^600^K/^600^R in HRB domain in Asia 1 isolates except for Yanaka strain while HRA domain was conserved

In other regions than the above described domains, common amino acid changes in all Asian isolates but different from Onderstepoort strain were found as ^317^K/^317^R, ^431^V/^431^I and ^556^S/^556^G. Group specific change(s) was found as ^395^V/^395^I in Asia 2 isolates while those were found as ^309^L/^390^F, ^429^K/^429^R, ^466^I/^466^L and ^607^S/^607^G in Asia 1 isolates, but strains Ac96I and Th12 have no amino acid difference at position 607 in compare to Onderstepoort strain. However, an unique amino acid to one or more strains in the same group was detected such as ^546^S/^546^G for M25CR and 011C, ^478^G/^478^C for 81ND and ^482^W/^482^L for 50Sc strains as shown in Fig 3.

### N-linked glycosylation sites and cysteine residues

The F protein of Asian isolates had seven potential glycosylation sites (Fig. 3). Four of them were recognized at positions 141-143, 173-175, 179-181 in the F2 region and 517-519 in the F1 region as reported previously [16-18,21]. Interestingly, the extra three potential glycosylation sites according to the consensus amino acid sequence for N-glycosylation site (N-X-S/T) were found in Asia 1 but not Asia 2 isolates, two at positions 62-64 and 108-110 in the signal peptide region were conserved in all Asia 1 strains while the site 605-607 in F1 region was found in some Asia 1 strains (50Sc, 81ND, 82Con and 83mLN). These seven glycosylation sites are shared by Taiwanese field isolates [22].

Cysteine amino acids are an important factor for the intra molecular disulfide bond and the steric structure of protein. As a result, a total 18 cysteine residues were detected in the F gene product; among them, fourteen residues were located at identical positions in all CDV strains (Fig. 3). Characteristic cysteine residues were located at positions 67, 116, 478 and 627.

### Phylogenetic analysis of amino acids of F genes

The identities between the amino acid and nucleotide sequences of Asia 2 and these of Onderstepoort were 91 % except for 50Con and 50Cbl (92 %), whereas identities between the amino acid and nucleotide sequences of Asia1 and Onderstepoort were 90 % and 91 %, respectively as shown in Table 1. Strains 66L and 009L, 011C and M25CR as well as strains 82Con and 83mLN showed 100 % identities in both amino acid and nucleotide sequences. The similarity of strain Th12 was 99 and 98 % to other Asia 1 strains in amino acids and nucleotides, respectively. While the similarity was 91 and 93 % to all Asia 2 isolates in amino acids and nucleotide sequences except for strains 50Con and 50Cbl amino acid identity was 92 % (Table 1).

**Table 1:** The identity of the deduced amino acid and nucleotide sequences of F genes of CDV Asian isolates.


**Virus**	**% of identity^a ^with:**										
	
	**007Lm**	**55L**	**66L^b^**	**M25CR^b^**	**50Con^b^**	**Ac96I**	**Th12**	**50Sc**	**81ND**	**82Con^b^**	**Ond**

007Lm		**99**	**99**	**99**	**99**	**92**	**91**	**91**	**91**	**91**	**91**
55L	99		**100**	**99**	**99**	**92**	**91**	**91**	**91**	**91**	**91**
66L^b^	99	99		**99**	**99**	**92**	**91**	**91**	**91**	**91**	**91**
M25CR^b^	99	99	99		**99**	**92**	**91**	**91**	**91**	**91**	**91**
50Con^b^	99	99	99	99		**92**	**92**	**92**	**92**	**92**	**92**
Ac96I	92	92	92	92	93		**99**	**99**	**99**	**99**	**90**
Th12	93	93	93	93	93	98		**99**	**99**	**99**	**90**
50Sc	92	92	92	92	93	99	98		**99**	**99**	**90**
81ND	92	92	92	92	93	99	98	99		**99**	**90**
82Con^b^	92	92	92	92	93	99	98	99	99		**90**
Ond	91	91	91	91	92	91	91	91	91	91	

^a ^The identity of the deduced amino acid (upper half) and nucleotide (lower half) sequences of F genes was found among CDV strains.

^b ^Values shown are identical for cases 66L and 009L, M25CR and 011C, 50Con and 50Cbl and 82Con and 83mLN.

**Figure 4:**
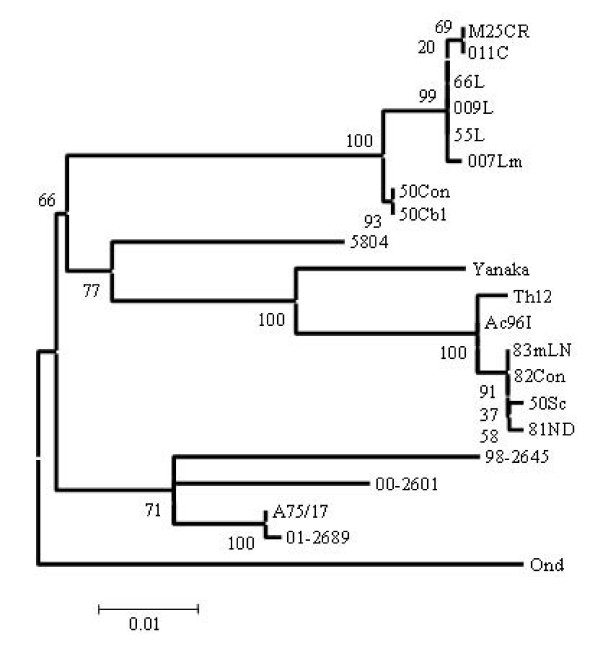
**Phylogenetic analysis of deduced amino acid sequences of F gene products of Asian isolates**. Accession numbers of CDV strains are shown in the legend of Fig. 1.

The Phylogenetic analysis of F genes revealed that Asia 2 strains clustered into four clades; clade 50Con and 50 Cbl, clade 007Lm, clade 011C and M25CR, clade 66L, 55L and 009L as shown in Fig. 4. On the other hand, Asia 1 isolates have five clades including clade Ac96I, clade Th12, clade 81ND, clade 50Sc, clade 82Con and 83mLN. Interestingly, Asia 1 isolates were appeared to be closer to the European (5804) strain than American strains (A75/17, 98-2645, 00-2601, and 01-2689) when the phylogenetic relationship of F gene (Fig. 4) was compared with that of H gene (Fig. 1). In addition to this, the identical strains in H gene sequences such as 66L, 009L, M25CR, 011C and 007Lm could be distinguished into three distant clades of Asia 2, and strains 82Con, 50Sc and 83meLN into two distant clades of Asia 1 by F gene sequences analysis as shown in Fig. 4.

**Figure 5:**
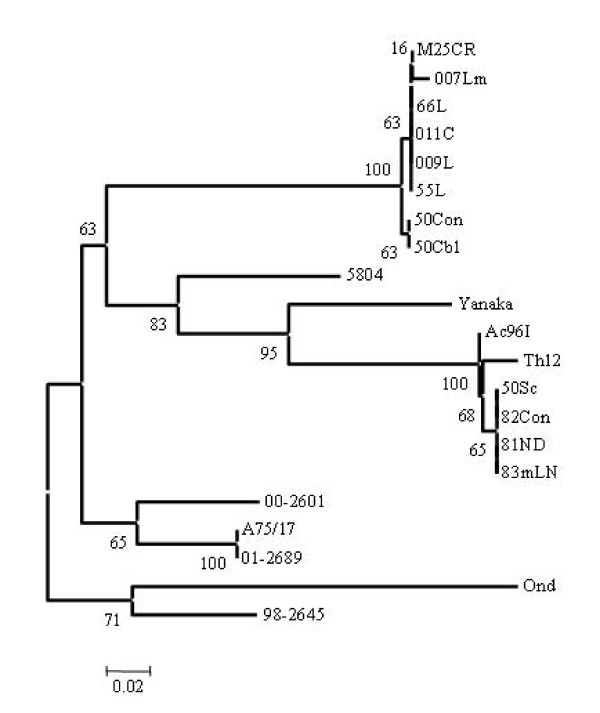
**Phylogenetic analysis of deduced amino acid sequences of signal peptide region in the F gene of Asian isolates of CDV**. Accession numbers of canine distemper viruses are the same as shown in the legend of Fig. 1.

The phylogenetic relationship among various CDV strains based on the deduced amino acid sequences of the signal peptide region (Fig. 5) showed similar but not identical classification to that of F gene (Fig. 4).

## Discussion

Although the presence of Asia 2 group of CDV was known previously by the sequencing and phylogenetic analysis of H gene [12], the characteristion of F gene or F protein of Asia 2 group had not been identified. In this study, the characteristic extra 27 nucleotides encoding extra 9 amino acids adding to the usual ORF of F gene and the usual 662 amino acids of F protein, respectively, were found for the first time in all Asia 2 isolates by sequencing analysis of F genes (Fig. 2 and 3). The extra 27 nucleotide sequences were identical and highly conserved among Asia 2 isolates. This fact indicates that Asia 2 isolates are easily distinguished from other CDV strains including Asia 1, American and European isolates, by this sequence.

The nucleotide change from ^4909^G to ^4909^T led to the appearance of new initiation codon from position 4908 upstream of the usual F gene ORF (Fig. 2). Previous studies have suggested that translation of F protein starts at the first initiation codon, AUG1, or at the second codon, AUG61, that locates in the signal peptide region [2,23]. Adding to these in-frame AUGs, a new AUG appeared in Asia 2 isolates. Thus, producing an unusual long signal peptide, depending on the translation initiation codon used in the case of Asia 2 isolates.

The signal peptide region cleavage is necessary for the F gene activation and expression on the cell surface [2]. However, many reports have indicated that the potential function of this region is indirectly affecting the fusion activity of F protein and thus potentially contributing to neurovirulence, although this function was different for CDV strains [2,6]. So, the frequent variation observed in signal peptide region among Asian isolates may account for viral pathogenesis. In addition to the four conserved N-linked glycosylation sites, three were found only in Asia 1 isolates. Previous studies suggested that N-linked glycosylation of viral envelope proteins (H of measles virus, F of Newcastle disease virus, F of Nipah virus or prM of Japanese encephalitis virus) plays a number of critical roles in the virus life cycle and in virulence mechanisms such as binding to cell surface receptors and protecting against antibody neutralization [24-28], also the glycosylation might play an important role in the cleavage dependent activation of the precursor F0 protein or in its transport to the sub-cellular region where the proteolytic cleavage occurs [29]. Our finding of different glycosylation sites of the F proteins suggested that these F proteins have different characters.

When compared to Asian isolates, European 5804 strain shared all N-glycosaltion sites with Asia 1 except for that at position 605-607; in contrast American strains have the common four glycosylation sites as Asian isolates in addition to one site 108-110 shared by strains A75/17 and 01-2689.

Interestingly, strains Ac96I and Th12 have the same amino acids as Asia 2 at position ^23^H and Th12 has unique amino acids at ^7^K and ^26^R. Also, Yanaka strain, Asia 1 isolate [9] has similar amino acids to Asia 2 isolates such as ^19^L, ^23^H, ^84^D and ^101^R as well as unique amino acids at positions ^57^F, ^67^G, ^94^F, ^95^I, ^98^V, ^104^K, ^116^R, ^126^M, ^130^L, ^151^N and ^513^G.

The genetic relationships shown in Fig 1 and 4 indicate that the field isolates form two separated lineages based on the deduced amino acids of F or H gene. Surprisingly, Asia 1 isolates appeared to be more closely related to European 5804 strain than to any other American strains by comparing the full F gene, while by H gene analysis, Asia 1 isolates were clearly distinguishable from European strain (Fig 1 and Fig. 4). Moreover, the phylogenetic analysis of the deduced amino acids of the signal peptide region of F genes is helpful for CDV classification giving a similar overview to that of the full F gene as shown in Fig 4 and 5.

## Conclusion

The phylogenetic analysis of F gene gives clear picture for the H gene identical CDV strains and the signal peptide region gives a remarkable differentiation between Asia 1 and Asia 2 isolates.

## Materials and methods

### Cells and Viruses

Vero.DogSLAMtag cells were established as described previously [30]. Cells were passaged and maintained in Dulbecco's modified Eagle's medium (D-MEM; autoclavable; Nissui Pharmaceutical Co. Ltd., Tokyo, Japan) supplemented with 10 % fetal bovine serum in a CO_2 _incubator at 37°C.

Fourteen CDV strains; 007Lm, 55L, 66L, 009L, M25CR, 011C, 50Con, 50Cbl, Ac96I, Th12, 50Sc, 81ND, 82Con and 83mLN, were isolated and propagated, one or a few times, in Vero.dogSLAMtag cells and stored at -80°C until use. Specimens were collected from diseased dogs as summarized in Table 2.

**Table 2:** Summarized data of CDV strains used in this study.


**Strain**	**Country**	**Organ**	**H accession number**	**F accession number**	**Reference**

007Lm	Japan	Lymph node	AB212730	AB474397	[31]
55L	Japan	Lung	AB295485	AB475099	Present study
66L	Japan	Lung	AB295486	AB475100	Present study
009L	Japan	Lung	AB252718	AB475101	[31]
M25CR	Japan	Cerebrum	AB475097	AB475097	Present study
011C	Japan	Cerebellum	AB252717	AB476401	[31]
50Con	Japan	Conjunctiva	AB295483	AB476402	Present study
50Cbl	Japan	Cerebellum	AB295481	AB476403	Present study
Ac96I	Japan	Intestine	AB212963	AB512286	[10]
Th12	Thailand	Brain	AB475098	AB509344	Present study
50Sc	Japan	Spinal Cord	AB295484	AB509345	Present study
81ND	Japan	Nasal discharge	AB295487	AB509341	Present study
82Con	Japan	Conjunctiva	AB295488	AB509342	Present study
83mLN	Japan	Mesenteric lymph node	AB295489	AB509343	Present study

### Sequencing of F and H genes of CDV and phylogenetic analysis

Vero.DogSLAMtag cells were infected with virus suspensions at MOI = 0.01 and incubated for 18 - 24 hours. When the CPE almost covered the cultures, total RNA was extracted using a MagExtractor™ RNA Extraction Kit (Toyobo Co., Ltd. Osaka, Japan) according to the manufacturer's instructions. Reverse transcription and PCR amplification (RT-PCR) were carried out using a ReverTra-Plus-™-RT-PCR Kit (Toyobo Co., Ltd. Osaka, Japan). The primers used were as follows: 5'ACTTGCCCGATCTCAAGCTA 3' and 5' ATGCTGGAGATGGTTT AATTCAATCG 3'. The forward represents nucleotides 4754 - 4773 of the M-F region in the positive sense and the reverse represents nucleotides 8969 - 8994 of the H-L region in the negative sense. The amplified PCR products (4240 bp) were purified by using a Gene Clean II kit (Biogene, Inc., USA) after agarose gel (0.7 %) electrophoresis, and sequenced directly using a Big Dye^® ^Terminator v.3.1 cycle sequencing kit (Applied Biosystems, Inc., CA, USA), with appropriate primers designed according to an overlapping strategy Table 3. The sequences were aligned by CLUSTAL W (1.83) Multiple Sequence Alignments (DDBJ) and phylogenetic analysis was carried out by the neighbor-joining method in Mega 3.1 program.

**Table 3:** Oligonucleotide primers used for RT-PCR amplification and nucleotide sequencing.


**Name**	**Sequence (5' - 3')^a^**	**Nucleotide position**	**Region**

*4754F*	*ACTTGCCCGATCTCAAGCTA*	*4754-4773*	M-F
5211F	AGTGTCTCAAAAGGAGCGAG	5211-5230	F gene
07-5379F	GGACCGACAGTGTCCATTAT	5380-5399	
5645F	GGCTACAGCTGCACAAATCA	5645-5664	
6210F	ACTGTCCCGAGGTATGTTGC	6210-6229	
6751F	TTGGCAGTCTCCTCAGTGTT	6751-6770	
CDV-HS1	AACTTAGGGCTCAGGTAGTCC	7054-7074	H gene
7661F	GTAGGCAAAGTTTTCCCCCT	7661-7680	
cdvhf1	TGTGTGTAGAAGAGAGCACTGT	7962-7983	
8691F	TTTATGACCCAATCCGGACG	8691-8710	
*CDVHS2*	*ATGCTGGAGATGGTTTAATTCAATCG*	*8969-8994*	H-L

^a ^Forward and reverse primers in *italic *used for RT-PCR.

## Competing interests

The authors declare that they have no competing interests.

## Authors' contributions

SS conducted most of this work under supervision of KK and KM. NC participated in isolation of CDV strains and N-TL participated in isolation of CDV strains and determined the sequences of H and F genes of strains 007Lm and Ac96I under supervision of RY. All authors have read and approved the manuscript.
